# Optimization of the *cry1Ah1* Sequence Enhances the Hyper-Resistance of Transgenic Poplars to *Hyphantria cunea*

**DOI:** 10.3389/fpls.2019.00335

**Published:** 2019-03-26

**Authors:** Chen Xu, Hui Wei, Like Wang, Tongming Yin, Qiang Zhuge

**Affiliations:** ^1^Co-Innovation Center for Sustainable Forestry in Southern China, Key Laboratory of Forest Genetics and Biotechnology, Ministry of Education, College of Biology and the Environment, Nanjing Forestry University, Nanjing, China; ^2^Jiangsu Provincial Key Construction Laboratory of Special Biomass Resource Utilization, Nanjing Xiaozhuang University, Nanjing, China

**Keywords:** *Bacillus thuringiensis*, codon optimization, *Hyphantria cunea*, poplar, transgenic plant

## Abstract

Increased expression of the insect control protein genes of *Bacillus thuringiensis* in *Populus* has been critical to the development of genetically improved plants with agronomically acceptable levels of insect resistance. *Bacillus thuringiensis* (*Cry1Ah1*) proteins with highly specific toxicity against *Hyphantria cunea* were screened using an indoor bioactivity assay to obtain hyper-resistant transgenic poplars. Then, the *Cry1Ah1* sequence was optimized and transformed according to the optimal codon in poplar using software of our own design (http://120.79.60.226:8080/u/chen/w/codonpoplar). A vector was constructed to transform poplar NL895. The *Cry1Ah1* gene was transformed to poplar NL895 and six transgenic lines were obtained. The expression and insecticidal effect of the *Cry1Ah1* gene in transgenic poplar were evaluated by PCR and ELISA, and the specific indoor activity and field insecticidal activity against *H*. *cunea* were compared with a control. We concluded that the insecticidal activity of the transgenic NL895 was significantly better against lower instar larvae of *H. cunea* than against higher instar larvae. The mortality and pupation rates clearly differed among the various instar larvae and between transgenic and non-transgenic poplar. We obtained poplar seedlings with hyper-resistance to *H*. *cunea* by screening Bt genes and optimizing their genetic sequence.

## Introduction

Poplar is an important fast-growing forest tree. The total area planted with poplar in China is among the largest in the world (Hu et al., 2017; Wang et al., 2018), at about eight million hectares (Lu, 2008; Tun et al., 2018). However, large-scale mono-species planting of poplar can lead to serious pest problems, especially from lepidopteran and coleopteran insects. Lepidopteran pests include *Hyphantria cunea* Drury*, Lymantria dispar* Linnaeus, *Apocheima cinerarius* Erschoff, *Malacosoma neustria* Motschulsky, and *Limacodidae* and *Notodontidae* moths (Wang et al., 2018). Chemical agents and bio-insecticides are not only costly but also tend to result in serious pollution. Therefore, breeding insect-resistant varieties and improving the insect resistance of forest trees have become inevitable choices when cultivating new varieties of poplar. Of all the insect-resistance genes, Bt has been the most widely studied in poplar (Peña and Séguin, 2001). Bt transgenic poplar with insecticidal effects has been developed (McCown et al., 1991). In 1993, China also reported transgenic *Populus nigra* transformed with the Bt *CrylAc* gene; the mortality rate of gypsy moth increased to 80–90% (Tian et al., 1993). In 2002, the State Forestry Administration approved a strain of transgenic poplar for commercialization in China; this was the world’s first commercialized transgenic tree species (Wang et al., 2018). During this period, insect-resistant transgenic *P*. *nigra* (Hu et al., 2001), *P*. *deltoides* (Ramachandran et al., 1993) *P*. *euramericana* (Wang et al., 1997), NL-80106 (*Populus deltoides* × *Populus simonii*) (Guo et al., 2004) and 741 poplar (Tian et al., 2000; Yang et al., 2003) were successfully cultivated, and there are now resources for continuously introducing new insect-resistant poplar varieties (Zuo et al., 2018).

The fall webworm *Hyphantria cunea* is a destructive pest that affects a number of ornamental trees and shrubs and several agricultural crops native to North America, where two morphs (red- and black-headed) are found (Cao et al., 2016). The black-headed morph was inadvertently introduced to Asia in 1945 and to Europe in 1946. Currently, it has spread to more than 32 countries worldwide (Sullivan et al., 2012). The extreme polyphagy of the fall webworm (Firidin et al., 2008), including high fecundity, short generation time, and high starvation resistance, facilitate its spread and potential to damage crops. The webworm is a polyphagous pest that inflicts US$1 billion in crop damage annually in China (Subramanian and Mohankumar, 2006). There are more than seven million hectares of poplar plantations in China. The large areas of pure forest and foreign tree species provide conditions for *H*. *cunea* to breed and spread, and in recent years it has become increasingly problematic.

*Bacillus thuringiensis* (Bt) isolates are being screened worldwide in search of new insecticidal genes. More than 320 *cry* genes grouped into 40 families have been isolated from Bt strains, with an insecticidal spectrum that extends over several invertebrate orders (Crickmore et al., 1998). Because the products of *cry1A* genes are reported to be toxic to lepidopteran pests, to improve insecticide expression 23 protoxin forms of Bt toxins were selected to test as toxins that act on *H*. *cunea*.

**Figure 1:**
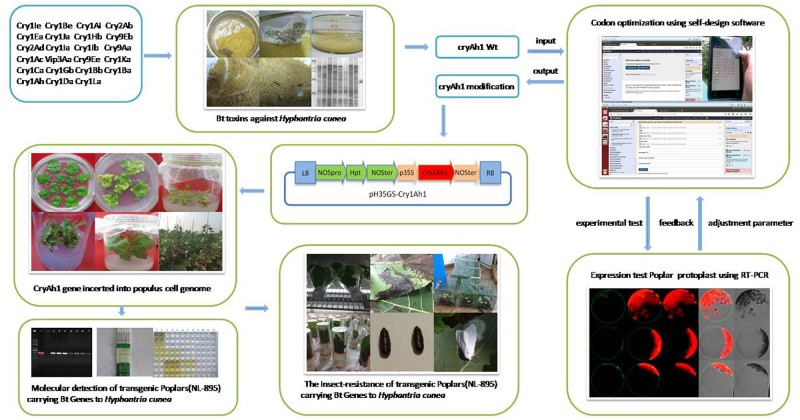
Flow chart of the transformation of transgenic poplars hyper-resistant to *Hyphantria cunea* using an optimized *cry1Ah1* sequence.

To increase gene expression to promote increased insecticide expression, *H*. *cunea* (*Cry1Ah1*) proteins with high toxicity were first screened using an indoor bioactivity assay. Then, our custom software was used to examine the coding sequence and produce synthetic genes, without changing the amino acid sequences. The demonstration of the utility of these genes for providing protection from insects has far-reaching implications for the future of insect-resistant woody plants and for the application of high-expression heterogonous gene design in woody plants (Perlak et al., 1991; Figure 1). Of particular importance and convenience is the highly efficient genetic transformation system coupled with efficient regeneration of poplar that is unsurpassed in other tree crops.

## Materials and Methods

### Plant Materials and Growth Conditions

Leaf disks were prepared from young *Populus deltoides* × *Populus euramericana* “Nanlin895” seedlings and pre-cultured for transformation (Zhang et al., 2015). The tested poplars were cultured by the leaf disc method with leaf explants. The seedlings were transplanted after 5 months of growth.

### Insect Strains

Insect bioassays were conducted to observe the responses of *H*. *cunea* larvae on the leaves of transgenic and control poplars. The first instar larvae of *H*. *cunea* were provided by the Research Institute of Forest Ecology, Environment and Protection, of the Chinese Academy of Forestry.

### Bt Screening Strains and BT Toxin Extract

Protoxin inclusion bodies were purified from transformed *Escherichia coli* from the general microbial collection of the Biotechnology and Biological Control Unit and randomly chosen to evaluate their insecticidal activity against worm larvae. The protoxin forms of Bt toxins were used in strains of Bt: Cry1Ie, Cry1Be, Cry1Ai, Cry2Ab, Cry1Ea, Cry1Ja, Cry1Hb, Cry9Eb, Cry2Ad, Cry1Ia, Cry1Ib, Cry9Aa, Cry1Ac, Vip3Aa, Cry9Ee, Cry1Ka, Cry1Ca, Cry1Gb, Cry1Bb, Cry1Ba, Cry1Ah, Cry1Da, and Cry1La^1^. For all experiments, the strains were provided by the Jie Zhang laboratory at the Institute of Plant Protection of the Chinese Academy of Agricultural Sciences (Gao et al., 2010; Li et al., 2014). Bt strains were routinely cultured on lysogeny broth medium. Protoxins were purified and solubilized as previously described (Lee et al., 1992). Solubilized protoxins were dialyzed against 50 mM Na_2_CO_3_ (pH 10). Protoxin concentrations were estimated by measuring total protein using the Bradford (1976) method, with bovine serum (BSA) as the standard, and percent toxin compositions were determined after the proteins had been separated by sodium dodecyl sulfate–polyacrylamide gel electrophoresis. All protoxin suspensions were stored at 20°C until use.

### Insect Bioassay

Bioassays were carried out with *H*. *cunea* using the diet-incorporation method with three replicates of 20 insects, each with appropriate controls (Meena et al., 2001). An aliquot of toxin solution was mixed with 15 g of diet at various concentrations. Neonates were fed an artificial diet and exposed to Bt protoxin (Gao et al., 2010). The toxin contents were based on the protein contents. The treated diet was then divided into three replicates and fed to neonates separately. Artificial diet without Bt protoxin was used as a control. Three replicates were set for each concentration, and 20 neonates were used for each replicate. The neonates were allowed to feed freely. Larval mortality was observed until 96 h. The mortality data were used to estimate the 50% lethal concentration (LC_50_) in terms of μg.g^-1^ diet as per the maximum-likelihood program.

### Codon Optimization Software Construction

We independently developed improved genetic modification software that was used to redesign the *cry1Ah1* sequence. The software, CodonPoplar (Supplementary Figure S1), is implemented in C with command-line options for the various parameters and is available from the author. It can be used online at http://120.79.60.226:8080/u/chen/w/codonpoplar. The redesigned sequences include localized regions of A+T richness resembling plant introns, potential plant polyadenylation signal sequences, ATTTA sequences, which have been shown to destabilize mRNA in other systems, and plant codon optimization. This software was used to synthesize the full *cry1Ah1* gene as follows:

(i) codon usage was altered to match certain features of host genes according to the optimal poplar codon;
(ii) the GC content was altered to match host genes;
(iii) known RNA instability motifs were avoided;
       (1) mRNA degradation signals were removed (ATTTA and ATTAA);
       (2) phylogenetic cleavage signals were removed (AATAAA, AATAAT, AATTAA, AACCAA, ATTA, ATTTA, ATAAAA, ATGAAA, AAGCAT, ATATAA, AATCAA, ATACTA, ATACAT, AAAATA, ATTAAA, AATTAA, AATACA, and CATAAA);
       (3) polyadenylation sequences were removed (AATAAT type, AATCAA type, AATGAA type, ATGGAA type, AATTAA type, TATAAA type, other types including ATGTAA, TGTGAA, AATGCT, GATATG, ATGCAA, AATGTG, AAAGAT, ATTAA, AATAAA, and AATAAT);
       (4) the intronic cleavage sequence (CATTG) contained in the coding region sequence was removed; and
       (5) continuous AT enrichment areas greater than four were modified;
(iv) potential RNA secondary structures near the translational start site were removed.

### Plasmid Construction and Transformation

The synthetic full-length sequences of the *cry1Ah1* genes were cloned into the Gateway entry vector pENTR/D-TOPO or pCR8/GW/TOPO (Invitrogen, Carlsbad, CA, United States), and transferred to the destination vector pH35GS (Kubo et al., 2005) by the LR reaction using LR Clonase II (Invitrogen). In the resulting plasmids, *cry1Ah1* was expressed under the control of the cauliflower mosaic virus (CaMV) 35S promoter. The plasmids were electroporated into *Agrobacterium tumefaciens* strain LB4404 and confirmed by agarose gel electrophoresis.

Putative transgenic plant lines were selected and propagated for PCR and real time (RT)-PCR. Following multiplex selection of transgenic poplar, the plantlets were grown on agar medium for 4 weeks in a confined culture room and moved to a greenhouse when there were four to five leaves on the top bud of the plantlet. After 3 days of acclimatization, the plantlets were transplanted into soil. The soil was then mixed with sterilized peat and perlite (2:1). During the first 2 weeks, water and vinyl membranes were used to maintain humidity.

### RT-PCR and Quantitative Real-Time Quantitative PCR (qPCR)

RT-PCR analysis of transgenic plants was performed by synthesis of first-strand cDNA with an enhanced avian RT-PCR kit using 5 μg of total RNA purified from a transgenic plant according to the manufacturer’s instructions (Sigma-Aldrich, St. Louis, MO, United States). The relative quantities of *cry1Ah1* transcripts in transgenic poplar plants were analyzed by qPCR performed using a StepOne real-time PCR system (Applied Biosystems, Foster City, CA, United States) and a QuantiFast SYBR green PCR kit (QIAGEN, Hilden, Germany). The poplar β-*actin* gene (GenBank Accession No. U60482) was used as an endogenous control in all RT-PCR assays. The nucleotide sequences of the forward and reverse primers for the *cry1Ah1* and β*-actin* genes were 5′-TCACTTCCCAAGCACATC-3′ and 5′ATCCTTCTCGGACAGACAA-3′, and 5’CTTCTCCTGTCGGTTTGTCG-3′ and 5′-TGGCAAATTTGAGGAGGTTC-3′, respectively, generating amplicons of 178 and 169 base pairs (bp), respectively. Total RNA extracted from 100 mg of leaf tissue was reverse transcribed into cDNA and used as a template in RT-PCR assays with *cry1Ah* and β*-actin* gene-specific primers. Reverse transcription was performed at 50°C for 10 min with an initial denaturation at 95°C for 5 min (for activation of hot-start *Taq* polymerase), followed by 40 amplification cycles comprised of 10 s denaturation at 95°C and a combined annealing and extension step at 60°C for 30 s in a 25-μl reaction mixture, according to the manufacturer’s instructions (QIAGEN). The relative values obtained from the quantitation of mRNA were expressed as 2^-ΔΔCt^, where ΔCt represents the difference between the cycle threshold (Ct) values of a target and the endogenous control (β-*actin*) in the same sample and ΔΔCt is the difference between the ΔCt value of a particular sample and that of the reference sample. The quantitative RT-PCR data represent mean values with standard error of three independent experiments with three replicates of the transgenic plant.

### Enzyme-Linked Immunosorbent Assay (ELISA)

Non-transgenic (CK) and transgenic Bt poplar samples were randomly selected. The procedures were performed using a Bt-cry1Ab ELISA kit (Agdia, Elkhart, IN, United States) with 0.5 g of each sample according to the manufacturer’s instructions. Total plant protein was measured by Coomassie blue staining of protein gels and each sample was measured five times.

### Insect Bioassay Testing

Six transformed transgenic poplar lines that showed relatively high expression levels of the target genes were selected for the insect-feeding experiment in the laboratory. Bt transgenic poplars and their corresponding non-Bt versions were exposed to first through sixth larval instar *H*. *cunea* to determine percent mortalities and times to pupation. The six strains and the non-transgenic poplar NL895 control were all set in the greenhouse in April. In June of that year, the first generation of *H*. *cunea* was placed in an artificial climate chamber at 28°C with 14 h of light. Uniform hatching and screening of healthy larvae were performed, and larvae of different ages were grouped and raised. The newly hatched larvae were raised to the test-insect age on leaves of the control poplar to obtain experimental insects at various ages, including 1-day-old larvae, which were used as test insects by raising them on the leaves of the control poplar for 1 day.

Using the indoor artificial group breeding method, from July to September second- to third-generation *H. cunea* and the third to fifth expanded leaves from the tops of the tested plants were collected and placed in 100-mL triangular flasks. Ten larvae were tested each day, using three flasks and three replicates. Since the larvae can climb, the flasks’ openings were sealed with gas-permeable gauze and then the flasks were inverted in an artificial climate chamber to ensure that the larvae could feed on the leaves normally. The leaves were replaced every 2 days until pupation, and larval growth and mortality were recorded.

### Insect Feeding on Transgenic Lines

Transgenic poplars were planted in the spring at a forest station nursery in Xuzhou, Jiangsu Province. Our transgenic poplars were granted a license for field experiments by the Biology Genetic Engineering Security Council of State Forestry Administration and were planted in controlled regions in March 2017. The wild goat cage experiment began in early September. In the natural case, a 50-cm tube of dense nylon mesh with a radius of 15 cm was placed on an upper branch of the plant. With indoor feeding, 1-day-old larvae were obtained as a test source. Each treatment started with 20 1-day-old larvae and 60 buds, and three replicates were performed. After the larvae were added, the ends of the nylon-mesh tube were closed and sealed, and larval growth status and mortality were recorded. The corrected mortality of the larvae at 6 and 12 days was determined. The SPSS statistics package was used to analyze the data. Mortality was corrected using Abbott’s formula:

Corrected mortality = 100 (Observed mortality - control mortality)/(100 - control mortality)

## Results

### Bt-Screened *H. cunea* With High Protein Toxicity Using the Indoor Bioactivity Assay

**Figure 2:**
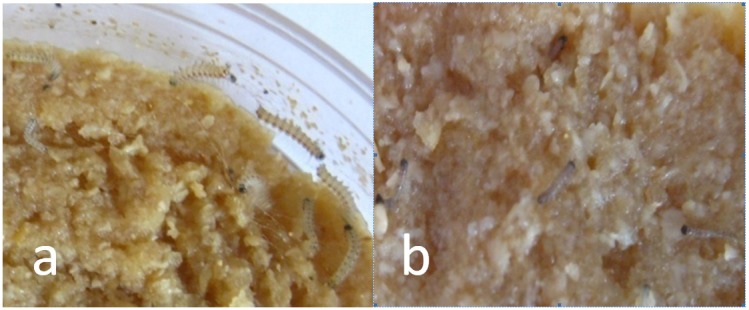
The larvae after Bt treatment with *Cry1Ah*. **(a)** The sides of the larval bodies in the control group were light yellow, and the larvae were very active at spinning and netting. **(b)** The larvae fed *Cry1Ah1* protein were dark, small, and stiff, and appeared dehydrated.

The bioassay results for each protein against the *H*. *cunea* larvae are shown in Table 1. When treated with 10 μg/g, the corrected mortality with *Cry1Ah1* reached 100%. With Cry1Ea and Cry1Ac, the protein toxicity against the American larvae was significantly lower and the corrected mortality reached 90%. Therefore, *Cry1Ah1* was selected as the target gene. In the control group, the sides of the larval bodies were light yellow, and the larvae were very active at spinning and netting. The bodies of larvae fed *Cry1Ah1* protein were dark, small, and stiff, and appeared dehydrated (Figure 2).

**Table 1:** Bioassay of 23 Bt toxins against *Hyphantria cunea.*

	Concentration		Corrected		Concentration		Corrected
Protein	(μg/g)	Mortality (%)	M (%)	Protein	(μg/g)	Mortality (%)	M (%)
Cry1Ie	10	6.67 ± 0.58	0	Cry9Aa	10	71.67 ± 0.58	69.09
	100	10.00 ± 1	1.8		100	100.00 ± 0	100
Cry1Be	10	6.67 ± 0.58	0	Cry1Ac	10	91.67 ± 1.53	90.91
	100	11.70 ± 0.58	3.63		100	100.00 ± 0	100
Cry1Ai	10	75.00 ± 4.58	72.73	Vip3Aa	10	13.33 ± 1.15	5.45
	100	100.00 ± 0	100		100	16.67 ± 1.15	9.09
Cry2Ab	10	80.00 ± 1	78.18	Cry9Ee	10	6.67 ± 1.53	0
	100	100.00 ± 0	100		100	18.33 ± 1.53	10.91
Cry1Ea	10	90.00 ± 0	89.09	Cry1Ka	10	11.70 ± 0.58	3.63
	100	100.00 ± 0	100		100	8.33 ± 0.58	0
Cry1Ja	10	15.00 ± 0	7.27	Cry1Ca	10	10.00 ± 1	1.8
	100	71.67 ± 2.08	69.09		100	73.33 ± 1.53	70.91
Cry1Hb	10	28.33 ± 2.52	21.82	Cry1Gb	10	13.33 ± 1.15	5.45
	100	11.70 ± 1.53	3.63		100	25.00 ± 2	18.18
Cry9Eb	10	20.00 ± 1.73	12.73	Cry1Bb	10	6.67 ± 0.58	0
	100	93.33 ± 1.53	92.73		100	1.67 ± 0.58	0
Cry2Ad	10	6.67 ± 2.31	0	Cry1Ba	10	10.00 ± 1	1.8
	100	13.33 ± 0.58	5.45		100	15.00 ± 1.73	7.27
Cry1Ia	10	8.33 ± 1.53	0	Cry1Ah	10	100.00 ± 0	100
	100	10.00 ± 1	1.8		100	100.00 ± 0	100
Cry1Ib	10	10.00 ± 0	1.8	Cry1Da	10	11.70 ± 0.58	3.63
	100	13.33 ± 0.58	5.45		100	16.67 ± 1.53	9.09
				Cry1La	10	26.67 ± 0.58	20
CK	0	8.30 ± 0.58	0		100	23.33 ± 1.15	16.36

^∗^*CK is the untreated control*.

### Gene Synthesis

Increased expression of insect-control protein genes of *B*. *thuringiensis* in *Populus* has been critical to the development of genetically improved plants with agronomically acceptable levels of insect resistance.

**Figure 3:**
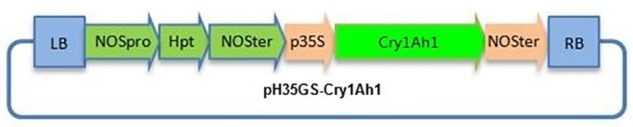
Schematic representation of the T-DNA region in the plasmid vector pH35GS-*cry1Ah*. LB T-DNA, left border repeat; p35sl CaMV35S promoter; NOSter, NOS terminator; NOSpro, NOS promoter; Hpt, hygromycin resistance gene; RB, T-DNA right border repeat.

**Figure 4:**
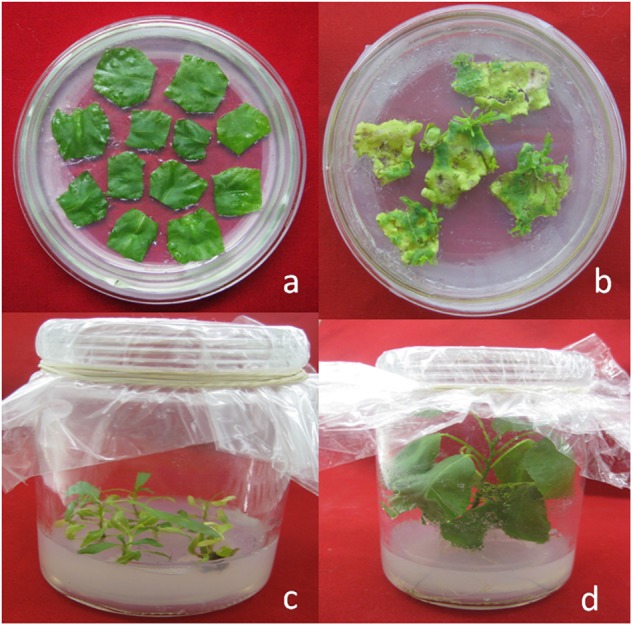
*Agrobacterium*-mediated transformation of poplar. **(a)** A typical pre-selection plate. **(b)** Tiny transgenic calli and shoot buds. **(c)** Transformants with healthy development of shoots and leaves. **(d)** Transgenic plantlets grown *in vitro*.

The gene encoding *cry1Ah1* was chemically synthesized and the codon was optimized to the full sequence of poplar cells. The synthetic gene sequence is shown (Supplementary Table S1). The gene was synthesized with 41.2% GC content and a 0.78 codon adaptation index (Table 2). The RNA secondary structure was removed and RNA instability motifs were avoided. There was no significant change in the GC contents of the two modified genes, and the minimum free energy of the RNA secondary structure of the modified genes was reduced from -239.84 to -291.20 kcal/mol, which made the transcribed mRNA more stable. Codon usage was adapted to the codon bias of the optimal poplar codon. The vector pH35GS-*Cry1Ah1* (Figure 3) was constructed and used to transform poplar clones (Figure 4).

**Table 2:** Comparison of the modified and wild-type *cry1Ah1* gene.

Parameter	Wild-type *cry1Ah1*			Modified *cry1Ah1*		
		Number			Number
	base	of bases	percentage	base	of bases	percentage
Base content	A	650	31.68%	A	616	30.02%
	C	337	16.42%	C	394	19.20%
	G	430	20.96%	G	451	21.98%
	T	635	30.95%	T	591	28.80%
Minimum free energy of mRNA secondary structure.	-239.84 kcal/mol			-291.20 kcal/mol		
RNA instability motifs	86			0		
RNA secondary structure (repetitive sequence)	12			0		
GC%	35.90%			41.20%		
CAI	0.27			0.78		
Relative expression RT-PCR	0.000275			0.032556		

### Results of Molecular Detection

**Figure 5:**
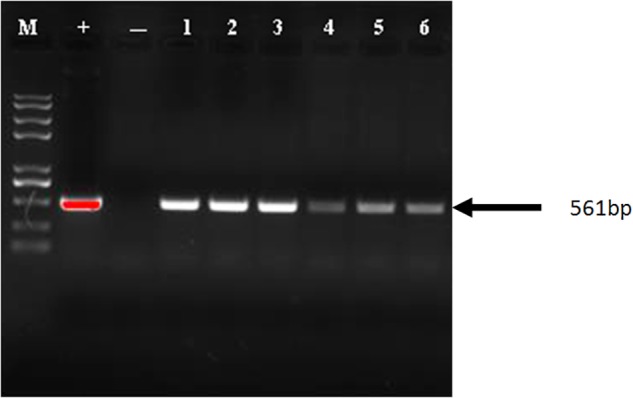
PCR analysis of the *Cry1Ah1* gene in transgenic plants. M, marker; +, plasmid; - indicate non-transgenic plant; L1-L6, transgenic plants.

The synthetic *cry1Ah1* poplar gene fragments were screened by PCR in six independent transgenic lines (Figure 5). PCR primers were used to amplify *cry1Ah1*. In transgenic plants, the primer pair amplified a band of 561 bp. RT-PCR showed that the modified genes provided enhanced expression (Supplementary Table S2). Expression of the new construct was greater than that of the wild-type gene, and the results showed improvement with respect to RNA stability and expression. The ELISA test of Bt protein expression in transgenic NL895 (Table 3) showed that the total protein content of all lines, including the controls, remained between 24.013 and 25.847 mg/g. Strain No. 6 had the highest toxic protein content, which reached 11500.01 ng/g, accounting for 0.0478% of the total protein expressed by this strain. The results showed that expression of the insecticidal protein was high. Toxic protein as a percentage of total protein content in all lines was at least 0.01%. In general, the higher the expression levels of toxic protein and the higher the percentage of total protein expression, the easier it is to make accumulation of toxins in the body of larvae play a role in killing them.

**Table 3:** ELISA test of Bt protein expression in transgenic NL895.

		Total protein	Toxic *Cry1Ah1*	Toxic protein as a
Transformed	Relative	content	protein content	percentage of the
lines	OD value	(mg.g^-1^)	(ng.g^-1^)	total protein (%)
Control	0.000	25.741 ± 1.32^a^	0.00 ± 0.00a	0.0000
No. 1	0.349	24.013 ± 0.54^b^	6987.91 ± 25.54^ab^	0.0291
No. 2	0.503	25.091 ± 1.43^bc^	10061.49 ± 17.19^bc^	0.0401
No. 3	0.439	25.847 ± 1.26^bc^	8787.91 ± 51.47^c^	0.0340
No. 4	0.467	24.140 ± 0.51^e^	9342.33 ± 31.10^cd^	0.0387
No. 5	0.153	24.077 ± 1.10^ef^	3057.83 ± 35.60^e^	0.0127
No. 6	0.575	24.059 ± 0.24^g^	11500.01 ± 102.23^f^	0.0478

*Following ANOVA, Duncan’s new multiple range method was used to compare the means. Means with different letters in the same column differ significantly at *P* < 0.05. The control was non-transgenic poplar*.

### Poplar Leaf Bioassay Based on Feeding by *H. cunea* Larvae

**Figure 6:**
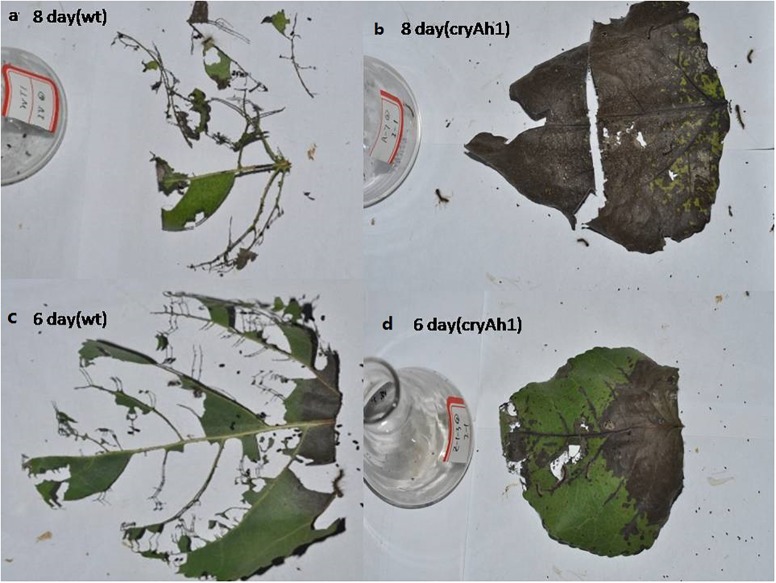
Poplar leaf bioassay of larvae after feeding for 6 or 8 days. Larvae fed **(a)** non-transgenic and **(b)** new *Cry1Ah1* transgenic poplar leaves for 8 days. Larvae fed **(c)** non-transgenic and **(d)** new *Cry1Ah1* transgenic poplar leaves for 6 days.

As shown in Table 4, the insecticidal activity of the transgenic NL895 was significantly better against lower instar larvae of *H*. *cunea* than against higher instar larvae in all poplar strains. The corrected mortality of the first to third instars increased gradually, was highest in the third instar larvae, and then gradually decreased in the fourth and fifth instars (Supplementary Figure S2). The corrected mortality of the first, second, and third instar larvae feeding on clone No. 6 was 54.2, 60.9, and 82.6%, respectively, and differed significantly from CK. The pupation rates of the first, second, and third instar larvae feeding on clone No. 6 were 7.4, 14.8, and 15.9%, respectively, and differed significantly from CK. The other five strains of transgenic poplar NL895 also differed from CK (Figure 6).

**Table 4:** Insecticidal activity of transgenic poplars with Bt on different instars of *Hyphantria cunea.*

Instar
larvae	Rate %	CK	No. 1	No. 2	No. 3	No. 4	No. 5	No. 6
1	6 Days corrected morality	7.1 ± 1.4	19.2 ± 6.6	15.4 ± 8.9	30.8 ± 7.4^∗^	26.9 ± 12.0	8.3 ± 3.6	42.3 ± 15.4^∗∗^
	12 Days corrected morality	12.4 ± 2.8	29.2 ± 5.3^∗^	37.5 ± 17.8^∗^	45.8 ± 20.0^∗^	41.7 ± 13.1^∗^	13.6 ± 5.9	54.2 ± 11.1^∗∗^
	Pupation	79.37 ± 4.7	20.7 ± 8.4^∗∗^	25.9 ± 12.8^∗∗^	22.2 ± 3.7^∗∗^	12.6 ± 7.8^∗∗^	55.5 ± 16.5	7.4 ± 2.1^∗∗^
2	6 d corrected morality	11.1 ± 4.1	24.0 ± 9.8	20.0 ± 16.0	40.0 ± 12.8^∗^	36.0 ± 10.7^∗^	4.1 ± 2.4	52.0 ± 15.7^∗∗^
	12 Days corrected morality	18.5 ± 3.2	34.8 ± 11.1^∗^	39.1 ± 8.8^∗^	47.8 ± 6.4^∗∗^	47.8 ± 5.7^∗∗^	4.5 ± 2.3	60.9 ± 6.4^∗∗^
	pupation	77.7 ± 6.8	29.6 ± 5.2^∗^	37.0 ± 9.4^∗^	29.6 ± 10.5^∗^	20.6 ± 7.5^∗^	51.8 ± 12.5	14.8 ± 5.7^∗∗^
3	6 Days corrected morality	7.4 ± 3.7	30.0 ± 8.6^∗^	36.0 ± 9.7^∗^	44.0 ± 10.7^∗∗^	48.0 ± 12.0^∗∗^	7.9 ± 2.1	60.0 ± 15.4^∗∗^
	12 Days corrected morality	14.8 ± 5.2	43.5 ± 16.0^∗∗^	52.2 ± 17.7^∗∗^	56.5 ± 20.0^∗∗^	52.1 ± 16.7^∗∗^	8.7 ± 2.1	82.6 ± 16.1^∗∗^
	pupation	81.5 ± 15.4	29.6 ± 6.4^∗^	37.0 ± 13.0^∗^	34.0 ± 11.4^∗^	29.6 ± 10.5^∗^	63.0 ± 8.6	15.9 ± 8.0^∗∗^
4	6 Days corrected morality	7.4 ± 2.1	24.9 ± 9.7	25.0 ± 15.5	29.1 ± 7.0	29.6 ± 5.0	8.0 ± 5.2	45.8 ± 10.1^∗^
	12 Days corrected morality	14.8 ± 6.4	27.2 ± 7.0	36.3 ± 8.6^∗^	36.3 ± 6.4^∗^	31.8 ± 11.6^∗^	13.1 ± 6.6	54.5 ± 13.5^∗∗^
	pupation	81.5 ± 15.1	40.7 ± 12.4^∗^	40.7 ± 14.5^∗^	37.0 ± 9.9^∗^	40.7 ± 14.4^∗^	51.9 ± 12.0	29.6 ± 10.3^∗^
5	6 Days corrected morality	3.7 ± 2.1	12.0 ± 6.4	14.2 ± 2.1	12.4 ± 5.9	12.4 ± 5.9	8.3 ± 4.5	20.8 ± 7.3
	12 Days corrected morality	17.1 ± 9.3	23.6 ± 3.5	22.7 ± 9.8	22.7 ± 5.4	18.1 ± 7.6	11.5 ± 7.7	31.8 ± 11.4^∗^
	pupation	80.2 ± 10.8	51.8 ± 18.5	48.2 ± 10.5^∗^	44.4 ± 14.2	51.8 ± 10.5	70.4 ± 16.5	37.0 ± 8.4^∗^
6	6 Days corrected morality	7.4 ± 3.7	8.3 ± 3.6	12.0 ± 9.5	10.0 ± 9.3	14.8 ± 7.7	4.0 ± 2.6	20.0 ± 11.3
	pupation	80.2 ± 14.4	76.7 ± 18.3	76.7 ± 26.7	63.0 ± 19.0	66.7 ± 15.4	79.3 ± 18.0	55.2 ± 15.6

^∗^*P < 0.05, ^∗∗^*P* < 0.01*.

Conversely, the pupation rates of the first to sixth instar larvae increased with age. The corrected mortalities of first to third instar larvae at 6 and 12 days increased with age, and was highest at the third instar, while the corrected mortalities of the third to sixth instar larvae decreased with increasing age. The insecticidal effect also decreased; when the sixth instar larvae were fed each strain of poplar, the insecticidal effect was no longer significant.

Table 5 shows the effects of transgenic Bt poplar on the development of *H*. *cunea*. Before 8 days of age, larvae fed on all strains successfully, however, the amounts consumed, growth rates, and pupal weights of the larvae fed on each strain differed. Strain No. 6 showed a significant difference (*P* < 0.05) at 6 days after feeding and 77 mg was consumed over 8 days of feeding (*P* < 0.01), compared with the control group, which consumed 183 mg. After 4 and 6 days of feeding on the leaves of No. 6, the body weights of the *H*. *cunea* larvae differed significantly from those of the control group (*P* < 0.05 and *P* < 0.01, respectively). The larvae fed on line No. 6 developed slowly, with an average body-weight gain of 3.3%, which differed significantly from the control (19.0%) (*P* < 0.01). The final pupal weight of those larvae that pupated also differed significantly compared with the control. In addition, the feed intakes and body weights of larvae fed on strains 2 and 4 began to show significant differences (*P* < 0.05) after 6 days, and the rates of weight gain and pupa weights also differed significantly (*P* < 0.05). Larvae fed on strains 2 and 4 consumed between 91.8 and 99.2 mg (96.3–104.0% of their body weight) over 8 days. There was a significant difference in the food intake (103.5 mg) over 8 days feeding on strain No. 3 by the white moth larvae. Additionally, strain No. 3 did not have a significant impact on the body-weight gain of the larvae. However, strain No. 1, which performed well in previous experiments, did not significantly affect the feeding intake or body-weight gain of insects in this experiment. Strains 6, 2, and 4 all inhibited development to varying degrees; the effect of strain 6 on the development of *H*. *cunea* was particularly evident.

**Table 5:** Insecticidal activity of transgenic poplars with Bt on the development of *Hyphantria cunea.*

									Mean weight	Pupal
Clone	Amount fed (mg)				Weight (mg)				growth rate (%)	weight (mg)
	2 Days	4 Days	6 Days	8 Days	2 Days	4 Days	6 Days	8 Days		
CK	35.9 ± 9.2	66.6 ± 14.8	115.2 ± 26.7	183.8 ± 35.1	76.0 ± 14.2	97.8 ± 10.4	120.6 ± 14.4	143.0 ± 12.9	19.0 ± 5.1	120.1 ± 14.0
No. 1	23.4 ± 6.9	68.2 ± 18.2	109.7 ± 35.4	150.9 ± 12.3	76.4 ± 5.9	86.4 ± 1.8	100.2 ± 7.3	119.4 ± 16.8	13.8 ± 6.9	101.5 ± 9.2
No. 2	23.3 ± 4.6	44.4 ± 15.3	77.6 ± 28.4^∗^	99.2 ± 36.6^∗^	72.4 ± 7.6	81.4 ± 13.8	90.8 ± 16.4^∗^	104.0 ± 26.5^∗^	11.4 ± 1.5^∗^	86.2 ± 14.0^∗^
No. 3	23.2 ± 4.1	41.5 ± 11.7	72.8 ± 34.1^∗^	103.5 ± 30.5^∗^	75.0 ± 8.4	85.4 ± 7.1	99.8 ± 7.5	116.2 ± 9.3	13.6 ± 1.6	96.2 ± 18.2^∗^
No. 4	26.7 ± 6.1	42.3 ± 6.5	73.7 ± 31.7^∗^	91.8 ± 20.1^∗^	76.4 ± 6.7	82.3 ± 8.8	87.5 ± 9.0^∗^	96.3 ± 11.9^∗^	7.4 ± 5.3^∗^	71.3 ± 9.8^∗^
No. 5	29.5 ± 5.0	71.3 ± 9.8	107.7 ± 32.2	154.9 ± 65.0	74.4 ± 10.2	93.8 ± 11.1	115.0 ± 11.3	133.8 ± 17.8	17.7 ± 5.0	106.4 ± 14.3
No. 6	20.3 ± 7.6	42.7 ± 5.4	66.9 ± 21.2^∗^	77.5 ± 23.2^∗∗^	76.3 ± 11.6	78.7 ± 7.0^∗^	80.5 ± 4.2^∗∗^	84.3 ± 2.5^∗∗^	3.3 ± 7.6^∗∗^	52.7 ± 5.4^∗∗^

^∗^*P < 0.05, ^∗∗^*P* < 0.01*.

### Insecticidal Activity of Genetically Modified Poplars Against *H. cunea* in the Field

Results from the specific field-cage experiments carried out at the beginning of July are shown in Table 6. Strain 6 showed the strongest effect, with 99.4% mortality of fifth instar insects. The cumulative mortality by the sixth instar was 99.8% and the pupation rate was 0. Strain 4 had the next strongest effect with 92.5% mortality of fifth instar insects and 99.8% mortality of sixth instar insects. There was no mortality of first instar larvae in the control group, in which the pupation rate was 78%. The pupation rate was less than 15% and mortality was greater than 60% in the third instar for all of the transgenic poplars. The insecticidal activity on *H. cunea* of genetically modified poplars was much higher in the field than in the laboratory. Field experiments were carried out directly on the leaves of transgenic poplar trees in bagging experiments. Indoor experiments mainly involved picking leaves and feeding them to larvae in an indoor constant-temperature incubator. Perhaps as a result, the leaves were dehydrated and Bt gene expression was weakened indoor.

**Table 6:** Insecticidal activity of transgenic poplars with Bt on *Hyphantria cunea* on the field.

Subclone	Instar (% mortality)						(% pupation)
	I	II	III	IV	V	VI	
CK	3.2 ± 8.3	6.4 ± 4.7	9.3 ± 7.6	18.4 ± 5.3	18.9 ± 3.9	19.1 ± 2.2	78.4 ± 24.9
No. 1	33.3 ± 5.9	46.9 ± 15.9^∗^	60.2 ± 4.0^∗^	78.1 ± 12.2^∗∗^	81.2 ± 10.4^∗∗^	82.6 ± 9.3	6.2 ± 10.6
No. 2	42.1 ± 7.2	78.8 ± 21.6^∗∗^	82.1 ± 11.1^∗∗^	88.1 ± 3.2	89.0 ± 3.4^∗∗^	91.9 ± 3.5^∗∗^	5.4 ± 3.4^∗∗^
No. 3	20.2 ± 3.8^∗∗^	64.1 ± 19.4^∗∗^	73.3 ± 12.7	79.5 ± 11.3^∗∗^	83.8 ± 9.9	83.2 ± 9.10^∗^	9.2+1.6
No. 4	46.9 ± 15.9^∗^	69.7 ± 34.6^∗∗^	72.4 ± 22.1^∗∗^	76.3 ± 10.4^∗^	92.5 ± 7.0^∗^	82.2 ± 3.3	10.4 ± 5.8^∗∗^
No. 5	34.2 ± 2.8^∗^	57.8 ± 18.5^∗∗^	79.4 ± 20.3^∗∗^	88.4 ± 13.6	80.7 ± 18.2	89.2 ± 1.2^∗∗^	2.0 ± 0.9
No. 6	31.4 ± 19.2	66.9 ± 16.3^∗∗^	80.2 ± 14.0^∗∗^	91.1 ± 3.5^∗∗^	99.4 ± 12.7	99.8 ± 0.2	0.0 ± 0.0

^∗^*P < 0.05, ^∗∗^*P* < 0.01*.

## Discussion

With the development of gene-design and gene-synthesis technology, software can now be used to design genes with greater expression. For the most part, gene-design software is simple. However, it is very difficult to apply to real plants. For example, it is important to remove the phylogenetic cleavage signals for plant transgenes, but most software does not do this. Therefore, we designed new gene-modification and synthesis software called CodonPoplar for poplars. Preliminary research to examine levels of heterogeneous gene expression in poplar was conducted.

The results of the mortality tests using wild larvae at different ages revealed that six lines of Bt-transgenic poplars had highly toxic effects on *H*. *cunea* under field conditions, and leaf-feeding by *H*. *cunea* decreased significantly. Moreover, the mean rate of pupation was below 10%. The third instar larvae had the highest resistance. *H*. *cunea* appears to be highly resistant to hunger. After eating the transgenic leaves, they significantly reduced their food intake, although they did not die immediately.

Kleiner et al. (1995) transferred the *Cry1Aa* gene to NC5339 poplar and fed transgenic leaves to gypsy moth and *Malacosoma* larvae. The mortality rates of the first- to third-instar larvae were significantly higher than those of the control group, but larval mortality after the third instar was not remarkable. Rao et al. (2000) fed gypsy moths Bt gene NL-80106 poplar leaves and found that the mortality of the first instar larvae was higher than that of the controls. We also found that the insecticidal effects of transgenic poplars on *H*. *cunea* gradually weakened with the growth of the larvae. However, a study of *H*. *cunea* larvae at different stages found that the mortality of the first instar larvae with effective transgenic lines was between 29.2 and 54.2%, while the corrected mortality of the third instar larvae was between 43.5 and 82.6%. We speculate that death was caused mainly by accumulation of toxic protein in the larvae, starvation, and other factors. Compared with second and third instar larvae, first instar larvae eat less, which may slow the accumulation of toxins in the larvae. Continuous feeding on leaves leads to accumulation of toxins in the larvae and is the direct cause of larval death. These larvae develop very slowly, and it takes 5–6 days to develop to the second instar. The amount of genetically modified leaves eaten was small. The Bt toxin protein content of the modified leaves was less than that of the Bt toxin extract used for Bt screen strains. In addition, larvae can withstand starvation for up to 10 days. Consequently, this led to a difference in results using the toxin extract for Bt screening strains (results not shown). The *H*. *cunea* larvae and pupae fed normal poplar leaves were significantly heavier than those fed transgenic Bt poplar leaves, and mortality was much higher on transgenic Bt poplar leaves than on normal poplar leaves.

Robison et al. (1994) fed gypsy moth larvae transgenic Bt gene NC5339 leaves and found that the larvae ate less than the controls and weight gain was severely delayed. Guo et al. (2011) fed transgenic Bt gene 741 leaves to *H. cunea*; after 21 days, the control group of larvae began to enter the sixth instar, while the larvae fed the transgenic leaves stayed at the second instar without molting; mean body weight and length were much lower than in the control group (Guo et al., 2011). Their study found that transgenic poplar also had a significant impact on the development of *H*. *cunea.* Our paper does not discuss other factors, such as the relationship between insect resistance and the age of transgenic *Populus* (Ren et al., 2018) or the stability of the toxin in plant tissues (Ren et al., 2017).

Many studies have proven that transgenic poplar has high resistance to *H. cunea*. The lethal effect of a transgenic single Bt gene line on *H. cunea* is 80–100% (Tian et al., 2000; Génissel et al., 2003; Ding et al., 2017, 2018; Wang et al., 2018). Although insect resistance has differed significantly among the various transgenic poplar varieties and strains, a positive correlation between toxic protein expression and the mortality of *H*. *cunea* larvae was reported (Zhang et al., 2016). In our study, we used a single Bt strain that had a remarkable insecticidal effect on *H*. *cunea*. Although expression of modified Bt genes in plants has improved greatly, with the advent of large-scale planting of transgenic plants the evolution of insect resistance cannot be ignored. To slow this process, previous researchers have used a multi-gene strategy (Yang et al., 2003, 2016; Klocko et al., 2014; Zhang et al., 2016; Ding et al., 2017). Additionally, an *H*. *cunea* population on transgenic poplar has been systematically surveyed for periods of 3 (Zhang et al., 2015) and 5 years (Zuo et al., 2018). A previous study found that the transformation of *Cry1Ac–Cry3A–NTHK1* genes in *Populus euramericana* “Neva” (Liu et al., 2016) led to mortality exceeding 60% for *H*. *cunea* and 100% for first instar larvae of the coleopteran *Plagiodera versicolora*; the author speculated that this was due to gene interactions (Liu et al., 2016).

In this study, a newly designed *Cry1Ah1* gene was successfully transferred into poplar and transgenic poplar lines with hyper-resistance to *H. cunea* were obtained. In the future, we may use the same method to select and modify new genes for transgenic poplar.

## Author Contributions

CX carried out the experimental work and prepared the first draft of the manuscript and figures. HW and LW provided critical inputs for the study as well as during preparation of the manuscript. QZ, CX, and TY designed the research, analyzed the results, and edited the manuscript and figures.

## Conflict of Interest Statement

The authors declare that the research was conducted in the absence of any commercial or financial relationships that could be construed as a potential conflict of interest.

## Footnotes

1 http://www.btnomenclature.info/

## Supplementary Material

FIGURE S1 The poplar gene optimization software CodonPoplar interface.

FIGURE S2 Mortality and pupation rates with *Cry1Ah1* for different instar larvae of *Hyphantria cunea*.

TABLE S1 *Cry1Ah1*-U is a codon optimization new sequence and *Cry1Ah1*-0 is wild sequence.

TABLE S2 The target gene relative transcript levels for RT-PCR.
