# Supplementary figures and images for: Optimization of the cry1Ah1 Sequence Enhances the Hyper-Resistance of Transgenic Poplars to Hyphantria cunea
Source: Front Plant Sci. 2019 Mar 26;10:335. doi: 10.3389/fpls.2019.00335 (PMC6443852; doi:10.3389/fpls.2019.00335)

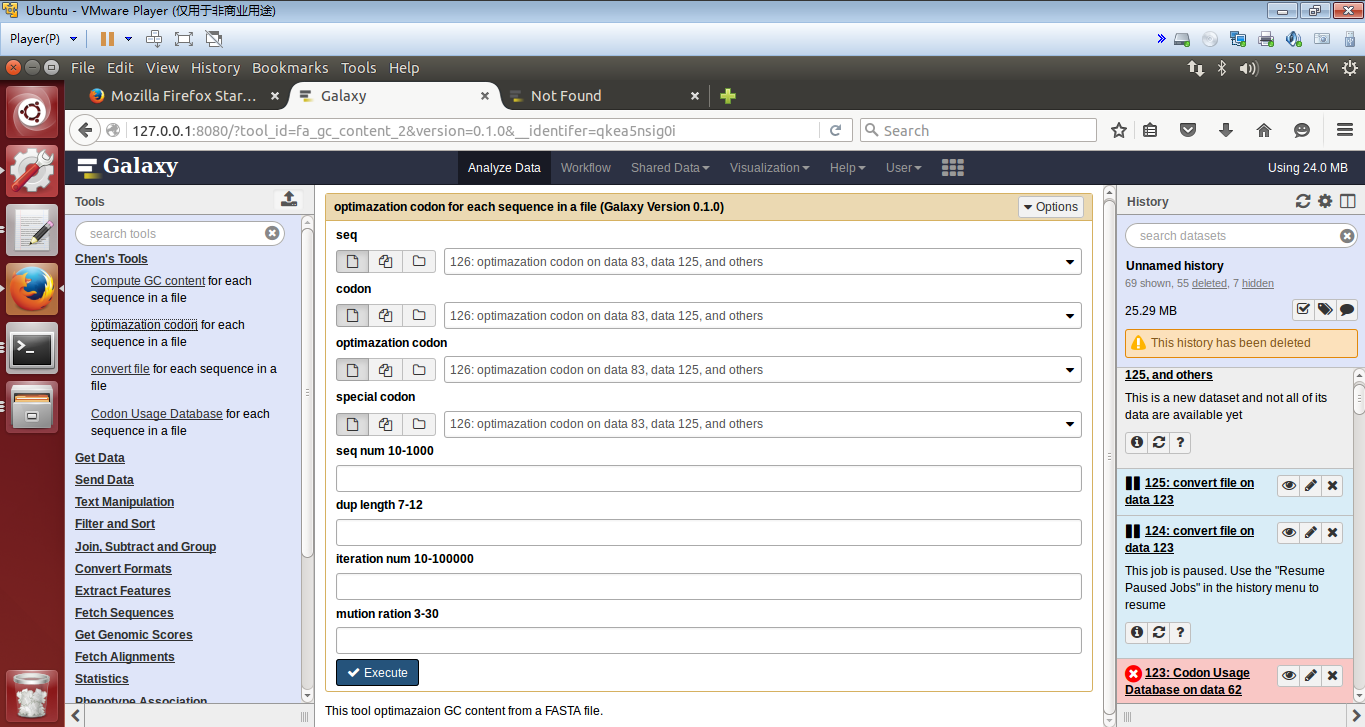

Supplement: FIGURE S1 — The poplar gene optimization software CodonPoplar interface. [file Image_1.PNG]

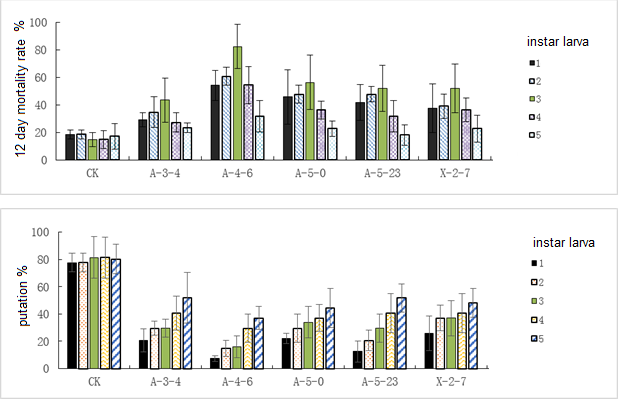

Supplement: FIGURE S2 — Mortality and pupation rates with Cry1Ah1 for different instar larvae of Hyphantria cunea. [file Image_2.PNG]
