# Supplementary material for: Optimization of the cry1Ah1 Sequence Enhances the Hyper-Resistance of Transgenic Poplars to Hyphantria cunea
Source: Front Plant Sci. 2019 Mar 26;10:335. doi: 10.3389/fpls.2019.00335 (PMC6443852; doi:10.3389/fpls.2019.00335)
Supplement: TABLE S1 — Cry1Ah1-U is a codon optimization new sequence and Cry1Ah1-0 is wild sequence. [file Table_1.docx]

Supplementary Table S1 Cry1Ah1-U is a codon optimization new sequence and Cry1Ah1-0 is wild sequence

cry1Ah1-0 ------------------------------------------------------------

cry1Ah1-U CACCTATTTTTACAACAATTACCAACAACAACAAACAACAAACAACATTACAATTACTAT

cry1Ah1-0 -----------------------ATGAAAAACAGTATCAAATTATCAGAACTTTGGTATT

cry1Ah1-U TTACAATTACATAAACCATGGCTGCTAAGAATTCTATCAAGCTTTCAGAACTTTGGTATT

** ** ****** * ************** *

cry1Ah1-0 TCAATGAAAGAAAATGGAGGTATTTTATGGAGATAGTGAATAATCAGAATCAATGCGTGC

cry1Ah1-U TTAATGAGAGAAAGTGGAGATATTTCATGGAGATCGTGAACAATCAGAATCAGTGTGTGC

* ** ** ** ** ***** ***** ******** ***** ** ***** ** ** ****

cry1Ah1-0 CTTATAATTGTTTGAATAATCCCGAAATCGAAATATTAGAAGGCGGAAGAATATCAGTTG

cry1Ah1-U CATACAATTGTCTTAACAATCCAGAAATTGAGATCTTGGAAGGAGGTAGGATCTCTGTGG

* ** ** *** * ** ***** ***** ** ** * ***** ** ** ** ** ** *

cry1Ah1-0 GTAATACCCCAATTGATATTTCTCTTTCGCTTACTCAGTTTCTTTTGAGTGAATTTGTCC

cry1Ah1-U GAAACACTCCTATCGATATTTCTTTGTCACTTACGCAATTTCTTTTGTCAGAGTTTGTTC

* ** ** ** ** ******** * ** ***** ** ********* ** ***** *

cry1Ah1-0 CAGGTGCGGGGTTTGTATTAGGATTAATTGATTTAATATGGGGATTTGTAGGTCCTTCCC

cry1Ah1-U CAGGTGCTGGTTTCGTGTTGGGACTTATTGATCTTATTTGGGGATTCGTTGGTCCATCTC

******* ** ** ** * *** * ** ** * ** ******** ** ***** ** *

cry1Ah1-0 AATGGGACGCATTTCTTGCTCAAGTGGAACAGTTAATTAACCAAAGAATAGCAGAAGCTG

cry1Ah1-U AATGGGATGCTTTTCTTGCACAGGTTGAGCAGCTTATCAATCAGAGAATTGCTGAGGCAG

******* ** ******** ** ** ** *** * ** ** ** ***** ** ** ** *

cry1Ah1-0 TAAGAAATACAGCAATTCAGGAATTAGAGGGAATGGCACGGGTTTATAGAACCTATGCTA

cry1Ah1-U TGAGAAATACTGCTATTCAAGAGCTTGAAGGAATGGCTCGTGTTTACCGTACTTATGCCA

* ***** ** ** ***** ** * ** ******** ** ***** * ** ***** *

cry1Ah1-0 CTGCTTTTGCTGAGTGGGAAAAAGCTCCTGATGACCCAGAGCTAAGAGAAGCACTACGTA

ccry1Ah1-U CAGCATTTGCTGAGTGGGAGAAGGCTCCAGATGATCCAGAGCTTAGAGAGGCTCTTAGAA

* ** ************** ** ***** ***** ******** ***** ** ** * *

cry1Ah1-0 CACAATTTACAGCAACTGAGACTTATATAAGTGGAAGAATATCCGTTTTAAAAATTCAAA

cry1Ah1-U CTCAATTCACTGCAACAGAGACTTATATCTCAGGTAGAATTTCAGTGCTTAAGATTCAAA

* ***** ** ***** ***** ** ** ** ***** ** ** * ** *******

cry1Ah1-0 CTTTTGAAGTACAGCTGTTATCAGTGTTTGCCCAAGCTGCAAATTTACATTTATCTTTAT

cry1Ah1-U CTTTTGAAGTGCAACTTCTTTCAGTGTTTGCTCAAGCTGCTAACTTGCATCTTTCTCTTC

******* ** ** ** * ******** ** ******** ** * ** * *** *

cry1Ah1-0 TAAGAGACGTTGTGTTTTTTGGGCAAAGATGGGGTTTTTCAACGACAACCGTAAATAATT

cry1Ah1-U TTAGAGATGTGGTGTTCTTTGGTCAAAGGTGGGGTTTCAGTACAACTACTGTAAACAATT

* ** ** ** ***** ***** ***** ******** ** ** ** ***** ****

cry1Ah1-0 ACTACAATGATTTAACAGAAGGGATTAGTACCTATACAGATTATGCTGTACGCTGGTACA

cry1Ah1-U ATTATAACGATCTTACTGAGGGTATTTCCACTTATACAGACTATGCTGTTAGATGGTATA

* ** ** ** * ** ** ** *** ** ******** ******** * ***** *

cry1Ah1-0 ATACGGGATTAGAACGTGTATGGGGACCGGATTCTAGAGATTGGGTAAGGTATAATCAAT

cry1Ah1-U ACACTGGTCTTGAAAGGGTTTGGGGTCCAGATTCACGTGATTGGGTTAGGTACAATCAGT

* ** ** * *** * ** ***** ** ***** * ******** ***** ** ** *

cry1Ah1-0 TTAGAAGAGAATTAACACTAACTGTATTAGATATCGTTGCTCTGTTCCCGAATTATGATA

cry1Ah1-U TTAGGAGGGAACTTACTCTTACTGTGCTCGATATCGTGGCTTTGTTCCCAAACTACGATT

* ** ** ** * ** ** ***** * ******** *** ******* ** ** ***

cry1Ah1-0 GTAGAAGATATCCAATTCGAACAGTTTCCCAATTAACAAGAGAAATTTATACAAACCCAG

cry1Ah1-U CTAGAAGATATCCAATTAGAACTGTTTCACAACTTACTAGAGAAATCTATACAAATCCAG

**** * ********* **** ***** ** * ** ******** ***** ** ****

cry1Ah1-0 TATTAGAAAATTTTGATGGTAGTTTTCGAGGCTCGGCTCAGGGCATAGAAAGAAGTATTA

cry1Ah1-U TTCTTGAGAATTTTGATGGTTCTTTTAGAGGTTCTGCTCAAGGTATTGAGAGATCTATTA

* * ** ************ **** **** ** ***** ** ** ** *** *****

cry1Ah1-0 GGAGTCCACATTTGATGGATATACTTAACAGTATAACCATCTATACGGATGCTCATAGGG

cry1Ah1-U GATCTCCACATCTTATGGATATTCTTAACTCCATTACTATCTATACTGATGCTCATAGAG

* ******* * ***** ** ** *** ** ** ** ** ** *********** *

cry1Ah1-0 GTTATTATTATTGGTCAGGGCATCAAATAATGGCTTCTCCTGTCGGTTTTTCGGGGCCAG

cry1Ah1-U GGTACTATTATTGGTCTGGTCATCAAATTATGGCTTCTCCAGTTGGATTCTCAGGTCCAG

* ** ***** *** ** ***** ** *********** ** ** ** ** ** ****

cry1Ah1-0 AATTCACGTTTCCGCTATATGGAACCATGGGAAATGCAGCTCCACAACAACGTATTGTTG

cry1Ah1-U AGTTTACTTTCCCACTTTACGGAACTATGGGTAACGCTGCTCCACAACAAAGAATTGTTG

* ** ** ** ** ** ** ** ** ***** ** ** ************ * *******

cry1Ah1-0 CTCAACTAGGTCAGGGCGTGTATAGAACATTATCCTCTACTTTTTATAGAAGACCTTTTA

cry1Ah1-U CTCAACTTGGTCAAGGTGTGTACAGAACTCTTTCTTCTACTTTTTACAGGAGACCCTTCA

******* ***** ** ***** ***** * ** *********** ** ***** ** *

cry1Ah1-0 ATATAGGGATAAATAATCAACAACTATCTGTTCTTGACGGGACAGAATTTGCTTATGGAA

cry1Ah1-U ATATTGGTATCAACAACCAGCAACTGTCTGTTCTTGATGGTACTGAGTTTGCTTATGGTA

* ** ** ** ** ** ** ***** *********** ** ** ** ******** ** *

cry1Ah1-0 CCTCCTCAAATTTGCCATCCGCTGTATACAGAAAAAGCGGAACGGTAGATTCGCTGGATG

cry1Ah1-U CATCATCTAACTTACCATCTGCTGTTTATCGTAAGAGTGGAACCGTTGATAGTTTGGATG

* ** ** ** ** ***** ***** ** * ** ** ***** ** *** **** *

cry1Ah1-0 AAATACCACCACAGAATAACAACGTGCCACCTAGGCAAGGATTTAGTCATCGATTAAGCC

cry1Ah1-U AGATTCCACCACAAAACAACAATGTTCCACCAAGACAAGGTTTTTCTCATAGACTTTCTC

* ** ******** ** ***** ** ***** ** ***** ** **** ** * *

cry1Ah1-0 ATGTTTCAATGTTTCGTTCAGGCTCTAGTAGTAGTGTAAGTATAATAAGAGCTCCTATGT

cry1Ah1-U ATGTGTCAATGTTTAGATCTGGTAGCTCTAGTTCTGTTTCTATTATTAGAGCTCCAATGT

**** ********* * ** ** **** *** *** ** ******** ****

cry1Ah1-0 TCTCTTGGATACATCGTAGTGCTGAATTTAATAATATAATTGCATCGGATAGTATTACTC

cry1Ah1-U TCTCATGGATTCATAGATCTGCTGAGTTTAACAACATTATTGCTTCAGACTCTATTACTC

**** ***** ** * ***** ** ** ** ** ** ** ** ** ********

cry1Ah1-0 AAATCCCTGCAGTGAAGGGAAACTTTCTTTTTAATGGTTCTGTAATTTCAGGACCAGGAT

cry1Ah1-U AAATTCCAGCTGTTAAGGGTAATTTCCTTTTTAACGGTTCTGTTATTTCTGGTCCAGGTT

**** ** ** ** ***** ** ** ******** ******** ***** ** ***** *

cry1Ah1-0 TTACTGGTGGGGACTTAGTTAGATTAAATAGTAGTGGAAATAACATTCAGAATAGAGGGT

cry1Ah1-U TTACTGGTGGTGATTTGGTGAGACTGAACTCATCTGGTAACAATATCCAAAACAGAGGTT

* ******** ** ** ** ** * ** *** ** ** ** ** ** ***** *

cry1Ah1-0 ATATTGAAGTTCCAATTCACTTCCCATCGACATCTACCAGATATCGAGTTCGTGTACGGT

cry1Ah1-U ATATTGAGGTTCCTATCCATTTTCCATCTACATCAACTAGATACAGAGTTAGAGTGAGAT

******* ***** ** ** ** *** ***** ** ***** ***** * ** * *

cry1Ah1-0 ATGCTTCTGTAACCCCGATTCACCTCAACGTTAATTGGGGTAATTCATCCATTTTTTCCA

cry1Ah1-U ACGCTTCTGTTACTCCAATTCATCTTAATGTTAATTGGGGTAATTCTTCTATTTTTTCTA

* ******** ** ** ***** ** ** *************** ** ******** *

cry1Ah1-0 ATACAGTACCAGCTACAGCTACGTCATTAGATAATCTACAATCAAGTGATTTTGGTTATT

cry1Ah1-U ATACTGTTCCAGCTACAGCAACTAGCTTGGATAACCTTCAATCTTCTGATTTTGGTTATT

* ** ** *********** ** ** ***** ** ** ** **************

cry1Ah1-0 TTGAAAGTGCCAATGCTTTTACATCTTCATTAGGTAATATAGTAGGTGTTAGAAATTTTA

cry1Ah1-U TTGAGTCTGCTAACGCTTTCACATCTTCACTTGGTAATATTGTTGGTGTTAGAAATTTTT

**** *** ** ***** ********* * ***** ** ** ***************

cry1Ah1-0 GTGGGACTGCAGGAGTGATAATAGACAGATTTGAATTTATTCCAGTTACTGCAACACTCG

cry1Ah1-U CTGGTACTGCTGGTGTTATTATTGATAGATTTGAGTTTATTCCAGTTACTGCTACTCTTG

*** ***** ** ** ** ** ** ******** ** ************** ** ** *

cry1Ah1-0 AGGCTGAATATAATCTGGAAAGAGCGCAGAAGGCGGTGAATGCGCTGTTTACGTCTACAA

cry1Ah1-U AGGCTGAGTATAACCTTGAGAGAGCTCAAAAGGCTGTTAACGCTCTTTTTACTTCTACTA

******* ** ** ** ** ***** ** ***** ** ** ** ** ***** ***** *

cry1Ah1-0 ACCAACTAGGGCTAAAAACAAATGTAACGGATTATCATATTGATCAAGTGTCCAATTTAG

cry1Ah1-U ACCAGCTTGGTCTTAAGACGAACGTTACTGATTATCATATTGATCAAGTTTCTAATCTTG

**** ** ** ** ** ** ** ** ** ******************** ** ** * *

cry1Ah1-0 TTACGTGTTTATCGGATGAATTTTGTCTG------------------------

cry1Ah1-U TTACTTGTCTTTCTGATGAGTTTTGTCTTTCCGAGAAGGATGAACTTTAATAA

**** *** * ** ***** ********
