# Supplementary material for: Optimization of the cry1Ah1 Sequence Enhances the Hyper-Resistance of Transgenic Poplars to Hyphantria cunea
Source: Front Plant Sci. 2019 Mar 26;10:335. doi: 10.3389/fpls.2019.00335 (PMC6443852; doi:10.3389/fpls.2019.00335)
Supplement: TABLE S2 — The target gene relative transcript levels for RT-PCR. [file Table_2.docx]

Supplementary Table S2 The target gene relative transcript levels for RT-PCR

|  | |
| --- | --- |
| NO. | Relative transcription level of Cry1Ah1-U |
| 1 | 0.034±0.001 |
| 2 | 0.012±0.0011 |
| 3 | 0.042±0.00078 |
| 6 | 0.038±0.001 |
| 10 | 0.019±0.006 |
| 12 | 0.041±0.0043 |
| 18 | 0.017±0.0057 |
| 23 | 0.053±0.001 |
| 24 | 0.037±0.0049 |
| CK+ | 0±0.0001 |

CK+ is No-transgenic poplar NL895.
